# Southern Medical Reports; Consisting of General and Special Reports, on the Medical Topography, Meteorology, and Prevalent Diseases, in the Following States: Louisiana, Alabama, Mississippi, North Carolina, South Carolina, Georgia, Florida, Arkansas, Tennessee, Texas

**Published:** 1850-07

**Authors:** 


*Southern Medical Reports; consisting of General and Special Reports, on the Medical Topography, Meteorology, and Prevalent Diseases, in the following States: Louisiana, Alabama, Mississippi, North Carolina, South Carolina, Georgia, Florida, Arkansas, Tennessee, Texas. To be published annually.* Edited by E. D. Fenner, M. D., of New Orleans, Member of the Am. Med. Association, etc., etc., etc. Volume I, 1849. New Orleans: B. M. Norman, 16 Camp Street. New York: Samuel S. & Wm. Wood, 261 Pearl St. 1850. 8 vo. pp. 472.

This volume is submitted in accordance with the plan announced in a *Prospectus* issued about a year ago, which was copied into this Journal. It is the first of a series of publications, to appear annually, which are to be devoted to reports by different individuals, on the medical topography, meteorology, prevalent diseases, etc., of the *Southern States.* In order that our readers may better appreciate the scope of the work, we quote the following from the Editor's introductory address, in which the objects of the enterprise are lucidly set forth:—

It surely will not be denied that the immense region of country which we have marked out as the scope of our observations, differs sufficiently in its prominent features of soil and climate, from the region lying north of it to be justly entitled the *Southern States,* as distinguished from the *North-*

*ern;* and it is also true that the various sections of this great Southern region differ very materially from each other in respect to climate, locality and geological formation. In like manner, a general distinction may be drawn between the prevalent diseases of the North and the South, as well as in the different sections of the South. Thus we may not only mark a difference in the general features of diseases in the various regions and sections, but they really call for a corresponding modification of treatment. Whoever expects to see the same remedies, administered in like doses, and in apparently similar conditions of the system, produce equally beneficial effects in these various regions and sections, will find himself egregiously mistaken. He must learn how to adapt his remedies and their doses to the peculiarities stamped upon disease by climate, locality, habit and mode of living, before he can ever become a successful practitioner.

In view of these considerations and many others now omitted, we shall endeavor to establish a *Magazine*, in which shall be recorded the practical observations and experience of all Southern Physicians whose energies are expanded beyond the bounds of self interest, and whose philanthropy would prompt them to contribute what they can to the relief of suffering humanity.

In our earnest endeavors to arouse the ambition and energies of Southern Physicians, far be from us the desire to see developed any *sectional feeling* beyond the strict proprieties of a laudable competition We have long been indebted to our brethren of the Northern States and of Europe for their profound researches and indefatigable labors; whilst they have long needed correct and faithful accounts of the ravages of Southern diseases upon the human constitution; and the best method of preventing them. They have been our preceptors in the elements and the principles of medicine; but we have obtained our knowledge of diseases from *personal observation.* Let us henceforth endeavor to reciprocate, with good feeling and proper courtesy, the obligations annually conferred by our brethren of the North and of Europe. We have a richer and more varied field for observation than they have, and, with equal industry, would be able to contribute more to the archives of medicine.

In commenting on the *Prospectus*, in a former No. of this Journal, we expressed our belief that the undertaking was worthy of all praise, and that the well known ability of Dr. Fenner as an observer, thinker, and writer, together with his experience as a medical editor, afforded an amply sufficient guaranty that the duty he proposed to himself would be faithfully and satisfactorily performed. Our pre-convictions are verified by an examination of the volume which has appeared. It contains little or nothing that is valueless, and much that must possess great practical interest for the medical profession of the South. For the latter it is specially designed, but Northern readers, who should desire to know something of the peculiarities of Southern diseases, and their relations to distinctive features of the South involved in Etiology, will do well to patronise Dr. Fenner's work. As another consideration, which we trust many of our readers will duly appreciate, we may suggest that an effort of this kind to develop and diffuse medical knowledge, and to arouse increased exertion by professional brethren in behalf of science and humanity, should not be regarded with indifference, and treated with neglect, even by those who do not directly participate in the benefits resulting therefrom. To issue a volume of this kind must require a considerable pecuniary outlay, and while we know that Dr. Fenner would not thank us for intimating a wish that his plan might prove a profitable speculation, we may be permitted to express a hope that, for the honor of the profession, the circulation of the work shall be sufficient to reimburse the cost of publication. In view of the consideration just suggested, the Southern Medical Reports has claims upon those of our Northern brethren who are desirous that useful laborers in the fields (not vineyards) of medical science, if they do not receive a reward, shall, at least, not be exposed to injustice; and who are ready to welcome and encourage every truly valuable addition to our native medical literature.

The present volume contains about forty articles, most of which were contributed, originally, for the work, and a portion selected from medical Journals. Some of the most valuable of the articles are from the pen of the Editor.

The price of the work is $5 per annum. Derby & Co., booksellers, of this city, will forward orders.

